# Group size influences maternal provisioning and compensatory larval growth in honeybees

**DOI:** 10.1016/j.isci.2023.108546

**Published:** 2023-11-23

**Authors:** Bin Han, Esmaeil Amiri, Qiaohong Wei, David R. Tarpy, Micheline K. Strand, Shufa Xu, Olav Rueppell

**Affiliations:** 1 State Key Laboratory of Resource Insects, Institute of Apicultural Research, Chinese Academy of Agricultural Sciences, Beijing 100193, China; 2 Delta Research and Extension Center, Mississippi State University, Stoneville, MS 38776, USA; 3 Department of Applied Ecology, North Carolina State University, Raleigh, NC 27695-7617, USA; 4 Biological and Biotechnology Sciences, Army Research Office, Army Research Laboratory, Research Triangle Park, Durham, NC 27709, USA; 5 Department of Biological Sciences, University of Alberta, Edmonton, Alberta T6G2R3, Canada; 6 Department of Biology, University of North Carolina, Greensboro, NC 27402, USA

**Keywords:** Entomology, Molecular biology, Evolutionary biology

## Abstract

Environmental variation selects for the adaptive plasticity of maternal provisioning. Even though developing honeybees find themselves in a protected colony environment, their reproductively specialized queens actively adjust their maternal investment, even among worker-destined eggs. However, the potentially adaptive consequences of this flexible provisioning strategy and their mechanistic basis are unknown. Under natural conditions, we find that the body size of larvae hatching from small eggs in large colonies converges with that of initially larger larvae hatching from large eggs typically produced in small colonies. However, large eggs confer a persistent body size advantage when small and large eggs are cross-fostered in small and large colonies, respectively. We substantiate the increased maternal investment by identifying growth-promoting metabolomes and proteomes in large eggs compared to small eggs, which are primarily enriched in amino acid metabolism and cell maturation. Thus, our study provides a comprehensive adaptive explanation for the worker egg size plasticity of honeybees.

## Highlights

• Honeybee queens adjust worker egg size in response to the colony environment
• Egg size influence on later weight depends on colony size
• Compensatory growth in large colonies negates small egg disadvantage
• Proteome and metabolome of large eggs are primed for growth and development

## Graphical abstract

**Figure:**
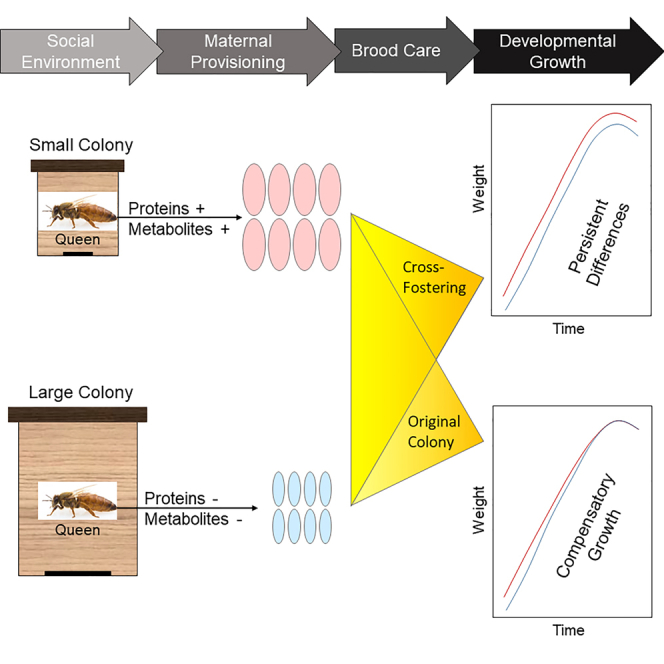

## Introduction

All parents face the fundamental life-history decision of how much to invest in individual offspring produced. Often this decision results from a concurrent optimization of offspring number and size,^1^^,^^2^ but trade-offs with survival or future reproduction are also prominent.^3^ The exact outcome of such trade-offs is influenced by the external environment that affects the costs and benefits to parent and offspring.^4^ Low-quality environments can increase the benefit of increased parental investment per offspring due to disproportional gains in offspring survival.^5^ This observation has led to the adaptive investment hypothesis, which explains that larger offspring or heightened parental care frequently occur in poor compared to favorable environments.^6^^,^^7^ Conversely, brood-caring helpers and cooperative kinship groups can lead to reduced parental investment in the anticipation of alloparental contributions that ensure the survival of less-provisioned offspring.^8^^,^^9^^,^^10^

Parental investment per offspring is readily quantified in females of oviparous species and many studies have characterized egg size plasticity in plants and animals, particularly birds and insects.^1^^,^^11^ Social insects, including the highly social honeybees of the genus *Apis*, are a notable exception to this abundance of studies, presumably because their colony organization and social protection mechanisms^12^ are thought to generate a highly predictable environment, which does not promote selection for egg size plasticity.^13^ However, honeybee queens, females that are solely specialized on reproduction, adjust the size of their eggs in multiple contexts: Queens produce larger eggs when ovipositing into specialized wax cells that predetermine the resulting offspring to be raised into new queens than when ovipositing into regular worker cells.^14^ Concomitantly, larvae hatching from larger eggs have a higher probability to be reared as new queens than larvae from smaller eggs when nurse bees are given the choice,^15^ and larger eggs also result in higher-quality queens, which might even have effects on the quality of offspring that these queens produce.^16^^,^^17^

Honeybee queens also increase the size of worker-destined eggs in response to small colony size and low food availability.^18^^,^^19^ This correspondence to the adaptive investment hypothesis^2^^,^^6^ is surprising because even in small colonies hundreds of workers are available for nursing the brood based on stored food resources^20^ and food shortages lead to demographic responses at the colony level that can buffer individuals.^21^ Thus, we set out to investigate the consequences of worker egg size plasticity for juvenile development in the context of small and large colonies. We identified growth advantages of larvae from large eggs that were dependent on colony size because the growth of larvae that hatched from small eggs in large colonies show compensatory growth. Our subsequent comparison of the small-versus large-egg maternal provisioning tactics with a combined metabolomics and proteomics approach demonstrated that large eggs contain not only more material than small eggs but are also better primed for growth and development.

## Results and discussion

Facilitated by their social organization, intense brood care, and protected hive environment, honeybee workers grow 1000-fold during five days from a 0.1 mg egg to a full-sized fifth larval instar.^22^ The highly social, homeostatically controlled hive environment thus may seem to make maternal plasticity in egg provisioning superfluous, in contrast to most other species.^6^ Yet, we discovered previously that honeybee queens reversibly adjust egg size according to perceived colony size; eggs were more than 10% smaller in colonies with 16,000–20,000 workers than in colonies with 500–700 workers.^19^

### Compensatory late larval growth in large colonies

**Figure 1:**
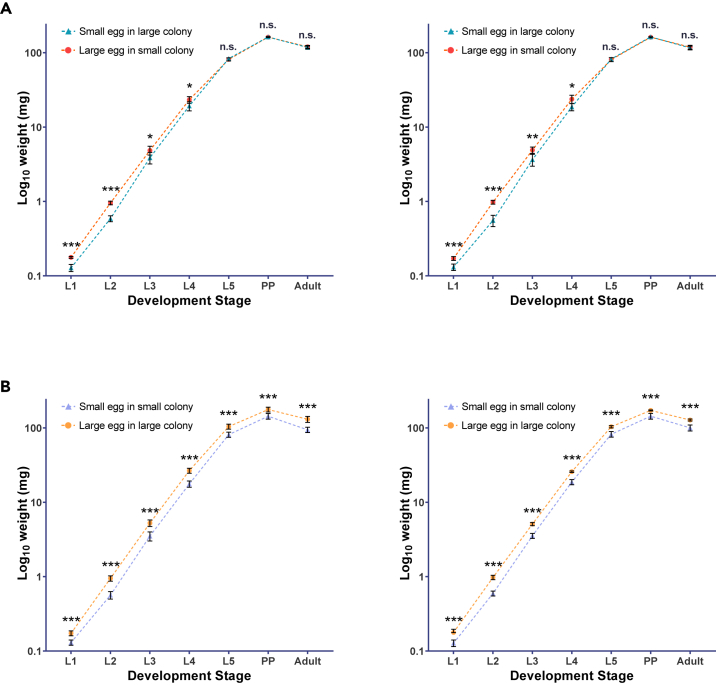
Growth Trajectories of Small and Large Egg Individuals in Original and Cross-Fostering Conditions Larger eggs translate into larger early development but larvae that hatch from small eggs in large colonies can compensate and grow to the same size at the final instar and adulthood (A). Under cross-fostering conditions, larvae hatching from small eggs in small colonies do not compensate by enhanced growth and remain smaller into adulthood than individuals that hatch from large eggs and develop in large colonies (B). For both experiments the left and right panel represent independent replicates. All weight differences were tested by pairwise t-tests and statistical supports are indicated by ∗ (p < 0.05), ∗∗ (p < 0.01), ∗∗∗ (p < 0.001), or n.s. (p > 0.05). Pools of 1^st^, 2^nd^, 3^rd^, 4^th^, and 5^th^ instar larvae (L1, L2, L3, L4, L5), pre-pupae (PP), and newly emerged bees (Adult) were used from five queens per group in each experiment. All data are presented as mean ± S.D.

To determine the developmental consequences of this difference, larvae from small and large eggs that developed in their own colony were weighed at each larval instar at 24-h intervals and as emerging adults (Figure 1A; Table S1). Larvae hatching from large eggs were significantly heavier as first instar (t_(5,5)_ = 7.3, p = 0.00008) and second instar (t_(5,5)_ = 10.8, p = 0.000005), and to a lesser extent also as third (t_(5,5)_ = 2.3, p = 0.048) and fourth (t_(5,5)_ = 2.5, p = 0.035) instar. However, after five days, the two groups reached similar in weights (t_(5,5)_ = −0.9, p = 0.42) and continued to display similar weights one day later (t_(5,5)_ = −0.5, p = 0.62) and as adults (t_(5,5)_ = 0.7, p = 0.50). These results were confirmed in a second replicate (Figure 1A; Table S1) and demonstrate that larger eggs convey an advantage in the developmental growth trajectory of young honeybees,^23^ which is common in insects^1^ and other organisms.^11^ The results also show that honeybee larvae hatching from small eggs in large colonies exhibit compensatory growth,^24^^,^^25^ facilitated by nursing workers that also stabilize the phenotype of developing workers.^26^^,^^27^ However, this growth might come at a survival cost, which could potentially explain the survival advantage of large over small eggs when reared in a common, large-colony environment.^18^

### Social environment limits compensatory growth in small colonies

In a reciprocal cross-fostering experiment of small eggs in small colonies and large eggs in large colonies, the initial weight differences persisted into adulthood (Figure 1B; Table S1). Large eggs developed into significantly heavier individuals than small eggs across all measurement time points (L1: t_(5,5)_ = 8.0, p = 0.00004, L2: t_(5,5)_ = 12.6, p = 0.000002, L3: t_(5,5)_ = 6.0, p = 0.0003, L4: t_(5,5)_ = 10.2, p = 0.00008, L5: t_(5,5)_ = 10.9, p = 0.000004, L6: t_(5,5)_ = 17.1, p < 0.000001, Adult: t_(5,5)_ = 10.8, p = 0.000005). These findings were also confirmed by matching results of a second replicate (Figure 1B; Table S1). The sharp contrast between the first and second experiments highlights the importance of colony size for brood care and the social environment in general.^28^^,^^29^ Nurse bees play a key role in honeybee development and the phenotype of the offspring,^26^^,^^30^ and the observed enhanced larval development in large colonies thus may be due to enhanced nursing. However, it remains to be tested whether the quality of honeybee nursing behavior changes with colony size or other potential benefits of large colony size, such as better homeostatic control,^12^^,^^31^ can explain our results. Although it is common for mothers to reduce maternal investment in many social species in response to alloparental helpers,^9^ our finding is remarkable because honeybees are swarm-founding species that always live in large groups with thousands of workers throughout their life cycle.^32^ By demonstrating individual-level consequences of the queens’ adjustment of egg size, we resolve the open question of the adaptive value of the observed egg size plasticity,^19^ although it remains to be studied how these individual effects translate to colony-level effects.

### Large eggs contain more metabolites and proteins related to growth and development

**Figure 2:**
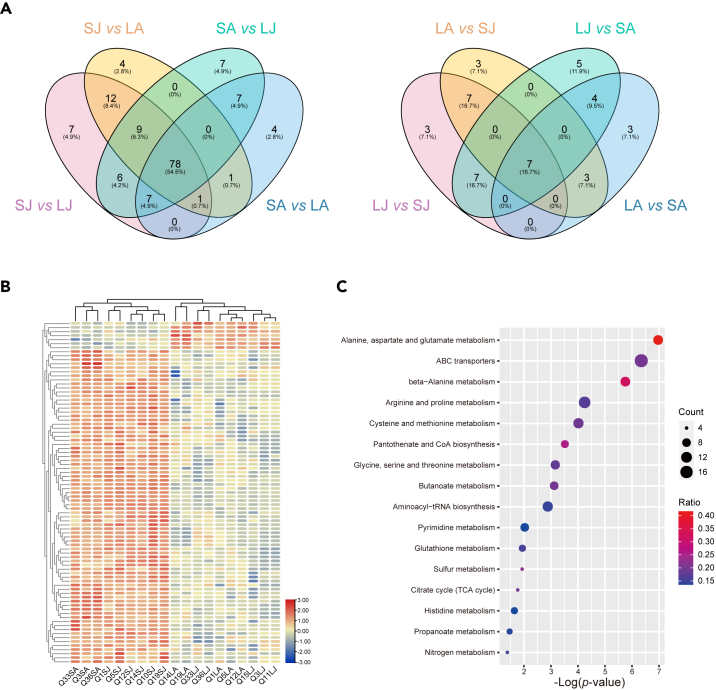
Metabolomic Differences between Small and Large Eggs Overlap analysis among different comparisons between small and large eggs indicated that more metabolites are over-abundant in large eggs compared to small eggs and that most of the differences are shared among comparisons (left Venn diagram). In contrast, few metabolites were more abundant in small compared to large eggs and the majority was not shared among all comparisons (right Venn diagram) (A). Clustering based on the abundance of core differential metabolites (indicated by the heatmap) unambiguously separated small and large eggs but did not completely resolve the temporal differences between July and August samples of large eggs (B). KEGG pathways enrichment analysis indicated 17 pathways (p < 0.05) with dot size symbolizing the number of metabolites and color representing the ratio (number of identified metabolites/total number of metabolites) for each pathway (C). LJ: large colonies in July; SJ: small colonies in July; LA: large colonies in August; and SA: small colonies in August.

To better distinguish adaptive from non-adaptive explanations of the observed egg size plasticity in honeybees, we compared small and large eggs in terms of metabolome and proteome content. Capitalizing on eggs collected during a reciprocal queen transfer experiment that was performed in July/August 2021 and indicated the reversibility of egg size adjustment in honeybees,^19^ we compared pools of 100 eggs per queen when they produced large eggs (in small colonies) and small eggs (in large colonies). Data from ultra-high performance liquid chromatography coupled with high-resolution mass spectrometry input to various databases yielded 390 compounds with an average peak area > 1E5. The four groups separated according to date (July vs. August 2021) and egg size (Figure S1). In July, 120 metabolites were significantly more abundant and 24 less abundant in large compared to small eggs. In the August samples, 98 more- and 17 less-abundant metabolites were found correspondingly. Core sets of 78 more- and 7 less-abundant metabolites were derived from the overlap of all four possible comparisons between large and small eggs across time points (Figure 2A). The overlap among more-abundant metabolites in large eggs (78/143) was significantly (Fisher’s Exact p < 0.0001) higher than the overlap among the more-abundant metabolites in small eggs (7/42). Based on these core sets, egg size divided the samples into two exclusive clusters (Figure 2B; Table S2). KEGG enrichment analysis identified 17 significantly enriched pathways in large eggs with the following top nine: “Alanine, aspartate and glutamate metabolism,” “ABC transporters,” “beta-Alanine metabolism,” “Arginine and proline metabolism,” “Cysteine and methionine metabolism,” “Pantothenate and CoA biosynthesis,” “Glycine, serine and threonine metabolism,” “Butanoate metabolism,” and “Aminoacyl-tRNA biosynthesis” (Figure 2C; Table S3).

The higher relative quantity of a number of metabolites in large eggs than in small eggs indicates that the flexible reproductive strategy of queens involves quantitative and qualitative differences. Large eggs not only provide more mass but also better nutrients, particularly in terms of amino acid metabolism, in contrast to houseflies^33^ and butterflies.^34^ Resource transfers from workers to queens in eusocial species can prevent resource-driven reproductive trade-offs^35^^,^^36^ and thus may facilitate the positive association between size and quality of eggs. Early embryonic development in honeybees is characterized by protein degradation and synthesis with amino acid intermediates,^37^ and the metabolome profiles suggest that these processes are up-regulated in large eggs. A higher abundance of metabolites related to ABC transporters also suggest that transport may be upregulated in larger eggs, but two ABC transporter gene sub-families in insects are also related to protein biosynthesis.^38^

**Figure 3:**
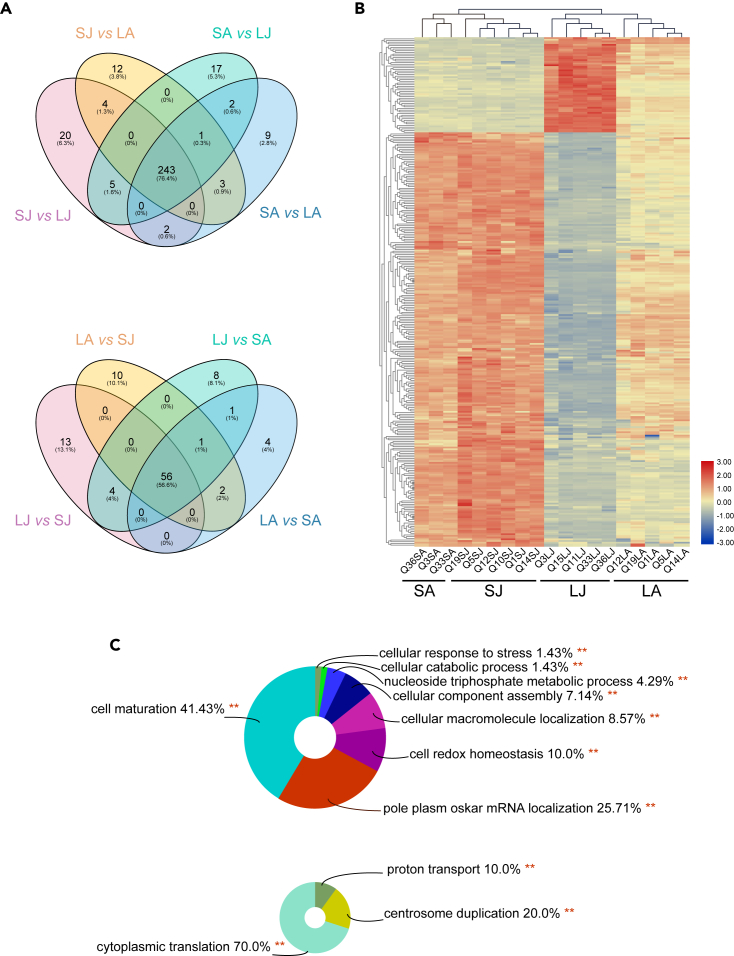
Proteome Differences between Small and Large Eggs Similar to our metabolomic results, a large number of proteins is more abundant in large relative to small eggs (A). The core of upregulated proteins in large eggs that are shared among all four possible contrasts between small and large eggs contained 243 proteins (left Venn diagram), more than 4x the number of proteins in the corresponding core set for proteins upregulated in small eggs (56, right Venn diagram). Based on these core proteins, perfect clusters according to egg size and sampling date form, except for one large egg sample from July that clustered with the large egg samples from August (B). GO terms enriched in the proteins upregulated in large eggs eight GO terms, while the corresponding list in small eggs contained three (C). LJ: large colonies in July; SJ: small colonies in July; LA: large colonies in August; and SA: small colonies in August.

Likewise, the LC-MS/MS-based proteome characterization indicated that more proteins were up-regulated in large relative to small eggs. Our four experimental groups clustered without overlap according to date and egg size based on 2040 identified protein groups, of which 1829 could be reliably quantitated (Figure S2). In July, 275 proteins were significantly more abundant and 72 less abundant in large compared to small eggs. In August, 260 more- and 64 less-abundant proteins were detected correspondingly. Based on overlap among all four possible contrasts between small and large eggs across both sampling dates, a core set of 243 more- and 56 less-abundant proteins was identified (Figure 3A; Tables S4, S5, S6, S7, and S8). The shared core represented a significantly (Fisher’s Exact p = 0.0002) higher proportion of the proteins that were more abundant in large eggs (243/318) than of proteins up-regulated in small eggs (56/99). The core proteome profiles were sufficient to separate all samples into two principal clusters of small and large egg samples, with secondary clustering mostly according to sampling date (Figure 3B).

**Figure 4:**
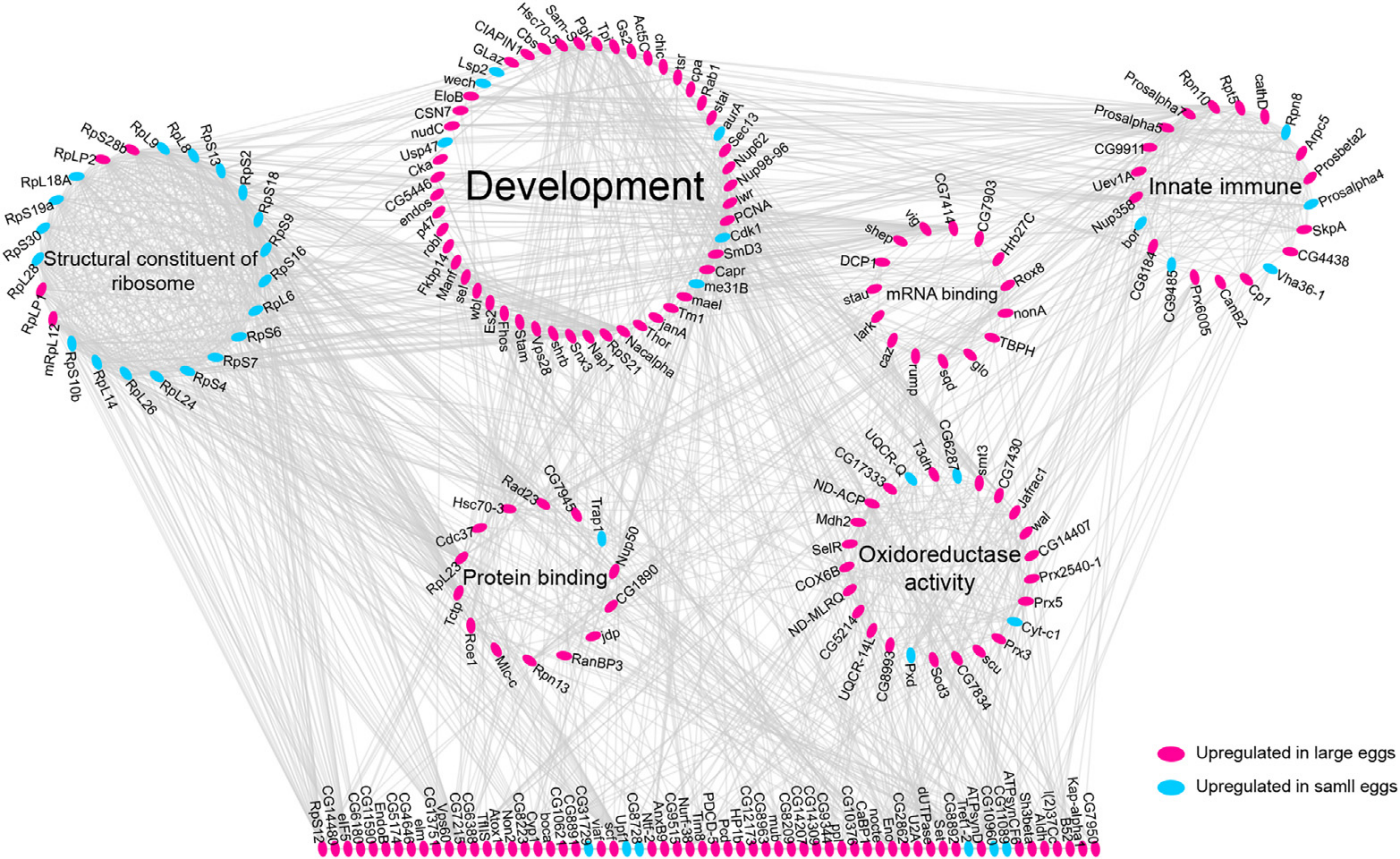
Interaction Analysis of Proteome Differences Between Small and Large Eggs The protein-protein interaction network analysis indicated six major clusters and numerous solitary proteins. One of the clusters (structural constituent of ribosome) was dominated by proteins upregulated in small eggs (colored blue), while the other five clusters and solitary proteins mostly contained proteins upregulated in large eggs (colored pink). The clustering thus reflects the overall results that large eggs are enriched in numerous proteins and also suggests that this enrichment is related to many biological functions because it includes several clusters of proteins and various solitary proteins.

GO analysis indicated that more abundant proteins in large eggs are enriched in eight biological processes: “Cell maturation,” “Pole plasm oskar mRNA localization,” “Cell redox homeostasis,” “Cellular macromolecule localization,” “Cellular component assembly,” “Cellular catabolic process,” Nucleoside triphosphate metabolic process, and “Cellular response to stress” (Figure 3C). In contrast, the proteins more abundant in smaller eggs were significantly enriched in “Cytoplasmic translation,” “Centrosome duplication,” and “Proton transport” (Figure 3C; Table S9). These results were complemented by KEGG pathway analyses that indicated “RNA transport,” “Carbon metabolism,” “Biosynthesis of amino acids,” “Protein processing in endoplasmic reticulum,” “Proteasome,” “Cysteine and methionine metabolism,” “Endocytosis,” “Oxidative phosphorylation,” and “Pentose phosphate pathway” to be more active in large eggs and “Ribosome” enriched in small eggs (Table S10). Protein−protein interaction (PPI) network analysis of all 299 core proteins connected 212 proteins successfully (Figure 4; Table S11) and revealed six functional groups: “Structural constituent of ribosome” (p = 3.36E-13), “Development” (p = 1.86E-07), “mRNA binding” (p = 6.33E-07), “Oxidoreductase activity” (p = 4.40E-04), “Innate immune system” (p = 1.40E-03), and “Protein binding” (p = 3.10E-03).

Thus, the proteome content of the differently sized eggs corroborates our interpretation of the metabolomic differences; in addition to their size advantage, large eggs are also qualitatively superior to smaller eggs, enriched with more proteins that function in cellular growth, maturation, and development. For example, the up-regulated core proteins contained insulin-like growth factor I (IGF-1) and eight translation initiation factors. IGF-1 controls metabolism^39^ and plays a key role in honeybee caste differences^40^ and division of labor.^41^ In contrast to adults,^41^ honeybee eggs display a positive association between IGF-1 and nutrient levels that is common in other species.^42^ These proteome differences presumably provide developmental benefits,^43^ enhancing embryogenesis and subsequent growth, which can thus explain the observed early developmental advantage of large eggs.^44^ Cellular transport processes, including cytoskeleton organization, are upregulated in ovaries that produce large eggs,^19^ providing a mechanistic explanation for the better provisioning of large compared to small eggs.

Overlap analysis of the core proteome with the proteome profiles of honeybee eggs aged 24, 48, or 72 h^37^ indicated no significant relationship (Fisher’s exact p = 0.73) between egg size and temporal differences, refuting the hypothesis that larger eggs simply undergo accelerated development. This result is corroborated by a similar lack of significant directional overlap between our core protein set and *Drosophila* proteins that change during the egg maturation process upon fertilization^43^ (Fisher’s exact p = 0.31, n = 16). The enrichment of proteins related to “Pole plasm oskar mRNA localization” is intriguing because *oskar* is not present in honeybees.^45^ Thus, the finding should be interpreted to indicate more generally an up-regulation of mRNA-binding proteins,^46^ which is also corroborated by our KEGG pathway and PPI results. The PPI analysis further indicated “Innate Immunity” as one major cluster containing five proteins up-regulated in small eggs and 16 up-regulated in large eggs. This mix suggests varying the regulation of different aspects of immunity and related processes, and it explains why GO and KEGG analysis did not identify this biological function as an overall difference between small and large eggs. The composition of honeybee eggs responds to maternal virus infection,^47^ although it is unclear whether transgenerational immunity against viruses exists in honeybees.^48^^,^^49^^,^^50^ Many proteins related to energy generation and homeostasis were up-regulated in larger eggs, indicating a more intensive metabolism,^51^ and our results also suggest that proteins involved in cell maturation and development are upregulated correspondingly. However, summarized in the GO terms “Proton transport,” “centrosome duplication,” and “ribosomal proteins,” some growth-related proteins were also found to be more abundant in small eggs. Potentially, these processes lay the foundation for compensatory larval growth after egg hatching under adequate conditions, which was observed when small eggs developed in large colonies.

### Conclusions

Overall, our study characterizes the qualitative differences that accompany egg size plasticity in honeybees, a species that should not be strongly selected for such plasticity because its advanced sociality provides a homeostatically regulated colony environment in which the offspring develop comparatively well-buffered from environmental fluctuations. The identified metabolomic and proteomic differences between small and large eggs suggest that there are no size-quality trade-offs in honeybee egg production. A well-defined and large set of metabolites and proteins are upregulated in large eggs, which are therefore larger and higher quality than small eggs. We further demonstrate the consequences of the different provisioning strategies; under normal circumstances, early developmental advantages of large eggs in small colonies are compensated by small-egg offspring that develop in large colonies to achieve similar adult body size. However, in offspring that lack their typical social environment, the initial developmental differences translate into different sized adults. The consequences of different body size in honeybee workers are unexplored, and it remains to be studied whether egg size plasticity of honeybee queens is a passive consequence of their tremendously plastic egg-laying rate or an evolved reaction norm with either current or past adaptive value.

### Limitations of the study

Our study was performed with unselected honeybees of the species *Apis mellifera* L., which is a diverse, cosmopolitan species and therefore we cannot exclude the possibility that our results are specific to the populations studied here. In addition, the proteomic and metabolomic work was done with samples from North America, while the growth experiments were performed in colonies from China. We cannot exclude the possibility that this could lead to a disconnect between these two parts of our study even though our previous study^19^ indicates that the observed effects are comparable in both populations.

## STAR★Methods

### Key resources table

**Table:** 

REAGENT or RESOURCE	SOURCE	IDENTIFIER
**Biological samples**		

Honey bee (*Apis mellifera*) eggs	Experimental apiaries of the University of North Carolina Greensboro and the Chinese Academy of Agricultural Sciences	N/A

**Chemicals, peptides, and recombinant proteins**		

Methanol	Thermo Fisher Scientific	Cat#A456-4
Acetonitrile	Thermo Fisher Scientific	Cat#A955-4
Formic acid	Thermo Fisher Scientific	Cat#28905
Trypsin	Promega Biotech	Cat#VA9000
Dithiothreitol	Sigma-Aldrich	Cat#D0632
Iodoacetamide	Sigma-Aldrich	Cat#A3221

**Critical commercial assays**		

Bradford Plus Protein Assay Kits	Thermo Fisher Scientific	Cat#A55866

**Deposited data**		

Egg proteomic data	Database: iProX	IPX0003758000

**Experimental models: Organisms/strains**		

Honey bee (*Apis mellifera*)	This paper	N/A

**Software and algorithms**		

SPSS v20.0	IBM	https://www.ibm.com/cn-zh/products/spss-statistics
GraphPad Prism v8.0.1	GraphPad Software	https://www.graphpad.com/
Xcalibur v3.0	Thermo Fisher Scientific	https://www.thermofisher.cn/order/catalog/product/OPTON-30965
PEAKS v8.5	Bioinformatics Solutions	https://www.bioinfor.com/download-peaks-studio/
Venny v2.1.0	Oliveros^52^	https://bioinfogp.cnb.csic.es/tools/venny
Metabolite Biological Role (MBROLE) v2.0	López-Ibáñez et al.^53^	http://csbg.cnb.csic.es/mbrole2
TBtools v1.1043	Chen et al.^54^	https://github.com/CJ-Chen/TBtools/releases.
KOBAS v3.0	Xie et al.^55^	http://kobas.cbi.pku.edu.cn/
CluePedia v2.5.7	Bindea et al.^56^	http://www.ici.upmc.fr/cluepedia/
Cytoscape v3.8.2	Shannon et al.^57^	https://cytoscape.org
Metascape v3.5.20230501	Zhou et al.^58^	https://metascape.org/
STRING v12.0	Szklarczyk et al.^59^	https://string-db.org/
SIMCA v14.1	Umetrics	https://shop.sartorius.com/ww/p/simca/UT-SS-1232
Compound Discoverer v3.2	Thermo Fisher Scientific	https://www.thermofisher.cn/cn/zh/home/products-and-services/promotions/industrial/compound-discoverer.html

### Resource availability

#### Lead contact

Further information and requests for resources and reagents should be directed to and will be fulfilled by the lead contact, Olav Rueppell (olav@ualberta.ca).

#### Materials availability

This study did not generate new unique reagents.

#### Data and code availability

The egg proteomic data and search results are deposited in the Proteome Xchange Consortium with the dataset identifier IPX0003758000 (https://www.iprox.cn/page/project.html?id=IPX0003758000).

Additional raw data are available in this paper’s supplemental information (Tables S1, S2, S3, S4, S5, S6, S7, S8, S9, S10, and S11; see below) and are publicly available as of the date of publication.

This paper does not report original code.

Any additional information required to reanalyze the data reported in this work paper is available from the lead contact upon request.”

### Experimental model and subject details

The studies were conducted on the Western honey bee (*Apis mellifera*) derived from commercial populations. All colonies were housed in the research apiary of the Institute of Apicultural Research in Beijing, China (for developmental studies) or the University of North Carolina Greensboro (metabolomic and proteomic studies). Standard management methods were employed to maintain the experimental colonies,^60^ with colony size and food status monitored and adjusted as necessary, but no other manipulations were applied during the experiments.

Three distinct colony sizes were established as previously described:^18^ ‘small’ colonies containing 500–700 worker bees housed in mating hives (nuclei) equipped with three half-frames of medium depth; ‘medium’ colonies containing 6,000–8,000 workers bees housed in 5-frame Langstroth hive boxes with standard frames; and ‘large’ colonies containing 16,000–20,000 worker bees in standard 10-frame Langstroth hives. These colonies were set up randomly from available source hives.

All queens used in the experiment were sisters raised from the offspring of a single mother to minimize genetic variation within experiments. Queen rearing followed standard methods, and two days before emergence each capped queen cell was introduced into a queenless ‘medium’ colony to emerge and for the new queen to mate naturally.^19^

### Method details

#### Larval development rate measurement

To investigate the effect of egg size on larval development, the developmental rate of larvae hatched from large and small eggs were first measured in their own colonies.

Ten sister queens were reared and randomly introduced into ‘large’ or ‘small’ colonies as described above. All queens were temporarily caged on empty comb for six hours to lay eggs. After 72 hours, 1st instar larvae were randomly collected and weighed with an analytical balance (AL204-IC, Mettler Toledo, Switzerland). Subsequently, additional larvae from the same cohorts were randomly selected and collected every 24 hours until cells were capped [L2 to pre-pupa (=PP)]. For L1 and L2, 50 larvae per queen were collected and combined for weighing, while 20 larvae were collected from L3 to PP and 20 newly emerged adults were collected.

To further test the effects of colony size on larval development, reciprocal cross-fostering experiments were performed in which eggs laid in ‘small’ colonies were randomly selected and exchanged with eggs laid in ‘large’ colonies. The weighing process was performed as described above.

Both the original rearing experiment and cross-fostering experiments were performed twice in 2022, once in May and once in July, with two sets of ten sister queens raised from a single mother, resulting in ten queens per treatment group. Due to the complex nature of this experiment, it was not performed blind. Statistical analyses were performed using SPSS v20.0, and results visualization were carried out using GraphPad Prism v8.0.1.

#### Egg metabolome analysis

To study the metabolomic difference between large and small eggs, an untargeted metabolomics approach was employed comparing four experiment groups: large colonies in July with small eggs (LJ), small colonies in July with large eggs (SJ), large colonies in August with small eggs (LA), and small colonies in August with large eggs (SA). These time points during the summer season were chosen to minimize seasonal variation.

Frozen egg samples (100 eggs per queen per time point) from each group were used for metabolome extraction. To extract small-molecule compounds, 1 ml of ice-cold 80% methanol/water (v/v) was added to each sample and homogenized with a bead mill homogenizer (Omni International, USA). The mixture was centrifuged at 12,000 rpm for 20 min at 4°C. The obtained supernatant was collected and filtered through a 0.22 μm membrane filter for subsequent ultra-high performance liquid chromatography–high resolution mass spectrometry (UHPLC–HRMS). To detect potential contamination, four blank control samples were prepared with 80% methanol alone and subjected to the above procedures. To evaluate the repeatability of the analytical system, a quality control (QC) sample was prepared by pooling 20 μl of all extracted egg samples.

UHPLC–HRMS analysis was performed on a Dionex UltiMate 3000 system (Thermo Fisher Scientific, Germany) coupled to a Q-Exactive mass spectrometer (Thermo Fisher Scientific). Buffer A (10 mM ammonium formate in 30% acetonitrile/water (v/v) and 0.1% formic acid) and buffer B (10 mM ammonium formate in 95% acetonitrile/water (v/v) + 0.1% formic acid) were used for gradient elution at a flow rate of 0.3 ml/min. Metabolites were separated using an ACQUITY BEH Amide column (150 mm × 2.1 mm, 1.7 μm; Waters, USA) with the following gradients: 0−1 min, 100% buffer B; 1−11 min 100%−30% buffer B; 11−11.5 min, 30%−100% buffer B; 11.5−13 min 100% buffer B. The eluted metabolites were injected into the mass spectrometer via a heated electrospray ionization source (Thermo Fisher Scientific). Two acquisition modes were adopted. The QC, egg, and blank control samples were analyzed in the full MS mode with the following settings: scan range = m/z 70–1,000; full scan resolution = 70,000; automatic gain control (AGC) target = 1E6; maximum injection time (MIT) = 50 ms. Six initial QC samples were injected in order to equilibrate the analytical system. Egg samples were injected in a randomized order to minimize systematic biases. Additionally, one QC injection was conducted after every five egg samples to monitor system stability and to correct peak areas of individual metabolite features.^61^ The injection volume was 2 μl for all samples. Parameters for MS/MS were as follows: scans resolution: 17,500; AGC target: 1 E5; maximum injection time: 50 ms; stepped collision energies: 15, 40, 60; loop count: 10. Different m/z range (70−205, 200−405, 400−605, 600−1,000, 70−1,000) was set for the QCs to maximize MS/MS spectra.

Raw data files were processed in the Compound Discoverer 3.2 (Thermo Scientific). Briefly, raw files were aligned using adaptive curve setting with 0.5 min retention time (RT) shift and 5 ppm mass tolerance. Unknown compounds were detected with the following settings: mass tolerance = 5 ppm; signal-to-noise ratio = 5; relative intensity tolerance for isotope search = 30%; peak intensity = 2E6 minimum then grouped with 5 ppm mass and 0.1 min RT tolerance. Peaks with a coverage value > 50 % and a relative standard deviation (RSD) value < 30 % for the six QC injections were retained for subsequent analysis. Metabolite annotation was performed by applying the search modules (5 ppm mass tolerance) of mzCloud and ChemSpider with three data sources, including Human Metabolome Database (HMDB), PubChem, and Kyoto Encyclopaedia of Genes and Genomes (KEGG).

Features generated by the Compound Discoverer were further filtered for an average peak area > 1E5 for QCs and for further multivariate and univariate analysis. Unsupervised principal component analysis (PCA) was implemented in SIMCA 14.1 (Umetrics, Umea, Sweden). Significantly different metabolites among experimental groups were determined by variable influence on projection (VIP ≥ 1) values derived from the OPLS-DA result, with a Benjamini–Hochberg corrected p-value (FDR ≤ 0.05). Venn diagrams were generated using Venny v2.1.0,^52^ which reflects the number of shared compounds in different sample groups.

To interpret quantitative differential compounds in biological terms, KEGG pathways enrichment was implemented using Metabolite Biological Role (MBROLE) v2.0.^53^ Over-representation analysis was computed using the cumulative hypergeometric distribution. These analyses were not performed blind.

#### Egg proteome analysis

To explore proteomic difference between large and small eggs, a label-free quantitative strategy was employed to compare variations between the four different experiment groups (LJ, SJ, LA, and SA).

The frozen egg samples (100 eggs per queen per timepoint) from each group were used for protein extraction by previously described method:^37^ Eggs were covered in lysis buffer (8 M urea, 2 M thiourea, 4% 3-[(3-cholamidopropyl) dimethylammonio]-1-propanesulfonate(CHAPS), 20 mM Tris-base, and 30 mM dithiothreitol (DTT)), thoroughly mixed and centrifuged at 15,000 rpm for 15 min at 4°C. After collection of the supernatant, proteins were precipitated with ice-cold acetone at -20°C for 30 min. The precipitate was collected by centrifugation (twice at 15,000 rpm for 10 min at 4°C) and discarding the supernatant. The pellet was dried at room temperature for 10 min and redissolved in 5 M urea. The protein concentration was determined using a Bradford assay, and the quality of extracted protein samples was checked by SDS-PAGE with Coomassie Blue staining.

Protein samples (200 μg) were reduced with DTT (final concentration 10 mM) for 1 hr, then alkalized with iodoacetamide (final concentration 50 mM) for 1 hr in the dark. Thereafter, protein samples were digested at 37°C overnight with sequencing grade trypsin (enzyme: protein (w/w) = 1:50). The digest was stopped by adding 1μl of formic acid and desalted using C18 columns (Thermo Fisher Scientific). The desalted peptide samples were dried and dissolved in 0.1% formic acid in distilled water, then quantified using a Nanodrop 2000 spectrophotometer (Thermo Fisher Scientific) and stored at -80°C for subsequent LC-MS/MS analysis.

LC-MS/MS analysis was performed on an Easy-nLC 1200 (Thermo Fisher Scientific) coupled Q-Exactive HF mass spectrometer (Thermo Fisher Scientific). Buffer A (0.1% formic acid/water) and Buffer B (0.1% formic acid and 80% acetonitrile in water) were used as mobile phase buffers. Peptides were separated using a reversed-phase trap column (2 cm long, 100 μm inner diameter, filing with 5.0 μm Aqua C18 beads; Thermo Fisher Scientific) and an analytical column (15 cm long, 75 μm inner diameter, filling with 3 μm Aqua C18 beads; Thermo Fisher Scientific) at a flow rate of 350 nl/min with the following 120 min gradients: from 3 to 8% buffer B in 5 min, from 8 to 20 % buffer B in 80 min, from 20 to 30% buffer B in 20 min, from 30 to 90% buffer B in 5 min, and remaining at 90% buffer B for 10 min. The eluted peptides were injected into the mass spectrometer via a nano-ESI source (Thermo Fisher Scientific). Ion signals were collected in a data-dependent mode and run with the following settings: scan range = m/z 300-1,800; full scan resolution = 70,000; AGC target = 3E6; MIT = 20 ms. For MS/MS mode, scans resolution = 17,500; AGC target = 1E5; MIT = 60 ms; isolation window = 2 m/z; normalized collision energy = 27; loop count = 10; dynamic exclusion = 30 s; dynamic exclusion with a repeated count = 1; charge exclusion = unassigned, 1, 8, > 8; peptide match = preferred; exclude isotopes = on. Raw data were retrieved using Xcalibur v3.0 software (Thermo Fisher Scientific).

The extracted MS/MS spectra were searched against the protein database of *Apis mellifera* (23,430 sequences, from NCBI) appended with the common repository of adventitious proteins (cRAP, 115 sequences, from The Global Proteome Machine Organization) using in-house PEAKS v8.5 software (Bioinformatics Solutions, Canada). The search parameters used were: ion mass tolerance = 20.0 ppm using monoisotopic mass; fragment ion mass tolerance = 0.05 Da; enzyme = trypsin; allow non-specific cleavage at none end of the peptide; maximum missed cleavages per peptide = 2; fixed modification = Carbamidomethylation (C, +57.02); variable modifications = Oxidation (M, +15.99); maximum allowed variable PTM per peptide = 3. A fusion target-decoy approach was used for the estimation of FDR and controlled at ≤ 0.01 both at peptide and protein levels. Proteins were identified based on at least one unique peptide.

Quantitative analysis of egg proteome from different experiment groups was performed using the PEAKS Q module. Feature detection was performed separately on each sample by using the expectation-maximization algorithm. The features of the same peptide from different samples were reliably aligned together using a high-performance retention time alignment algorithm. The label-free quantitation was applied based on extracted ion chromatograms and normalized using total ion chromatograms. These analyses were not performed blind.

Venn diagrams were again generated using Venny v2.1.0, which reflects the number of shared proteins in different sample groups. All the quantified proteins were used for PCA by SIMCA v14.1 software (Umetrics).

A heatmap of the overlapping differentially expressed proteins was created and clustered using TBtools v1.1043 software.^54^ The clustering was performed with Euclidean distance and complete method.

#### Bioinformatics analyses

In order to provide a better interpretation of the experimental results, honey bee proteins were mapped to *Drosophila melanogaster* for further bioinformatics analyses using KOBAS v3.0.^55^ Proteins of interest were uploaded in fasta format, *Drosophila melanogaster* was selected as target species, and sequence similarity mapping was conducted with default cutoffs (BLAST E-value < 1E-5 and rank ≤ 5).

To perform functional enrichment analysis of differentially expressed proteins, the ClueGO + CluePedia v2.5.7,^56^ a Cytoscape v3.8.2^57^ plugin, was employed to enrich functional categories based on biological processes of Gene Ontology (GO). Two-sided hypergeometric test (enrichment/depletion) with p-value ≤ 0.05 was used followed by a Bonferroni correction. The GO tree interval was set between 3 to 8 with a minimum of five genes and 1% of all genes. Kappa score ≥ 0.4 was applied to generate term-term interrelations and functional groups based on shared genes between the terms. KEGG pathway enrichment analysis was done by Metascape^58^ with default settings: min overlap = 3; p-value cutoff = 0.01; min enrichment = 1.5.

For the exploration of functional protein connections involved in biological activities, protein−protein interaction (PPI) networks were constructed by STRING v12.0.^59^ A full STRING network was built with medium confidence (0.4) and medium FDR (0.05). The PPI networks were visualized using Cytoscape v3.8.2.

### Quantification and statistical analysis

For the analysis of larval growth, the weight data were analyzed by two-tailed unpaired student t tests for each larval stage because the larvae were weighed destructively and thus a repeated measures approach was inappropriate. Significantly different metabolites between pairs of experimental groups were determined by variable influence on projection (VIP ≥ 1) values derived from the OPLS-DA result, Benjamini–Hochberg corrected p-value (FDR ≤ 0.05). The label-free quantitation was applied for protein quantification based on extracted ion chromatograms and normalized using total ion chromatograms. These data were analyzed with pairwise ANOVAs to specifically target the four pairwise comparisons between small and large eggs (LJ vs. SJ, LJ vs. SA, LA vs. SJ, and LA vs. SA), adjusting for multiple comparisons with the Benjamini–Hochberg correction (FDR ≤ 0.05). Sample sizes were determined in advance based on the results of previous results,^18^ and all available data were included in the analyses.

## Supplemental information

Document S1. Figures S1 and S2

Table S1. Growth trajectories of small and large egg individuals in original and cross-fostering conditions, related to Figure 1

Table S2. Core differential metabolites between small and large eggs, related to Figure 2B

Table S3. KEGG pathway enrichment results of core differential metabolites between small and large eggs, related to Figure 2C

Table S4. Differentially expressed proteins between eggs collected form SJ (small colonies in July) and LJ (large colonies in July), related to Figure 3A

Table S5. Differentially expressed proteins between eggs collected form SJ (small colonies in July) and LA (large colonies in August), related to Figure 3A

Table S6. Differentially expressed proteins between eggs collected form SA (small colonies in August) and LJ (large colonies in July), related to Figure 3A

Table S7. Differentially expressed proteins between eggs collected form SA (small colonies in August) and LA (large colonies in August), related to Figure 3A

Table S8. Core differentially expressed proteins between small and large eggs, related to Figure 3B

Table S9. Gene Ontology enrichment results of core differentially expressed proteins, related to Figure 3C

Table S10. KEGG pathway enrichment results of core differentially expressed proteins, related to Figure 3

Table S11. Protein–protein interaction results of core differentially expressed proteins, related to Figure 4
