# Supplementary material for: Group size influences maternal provisioning and compensatory larval growth in honeybees
Source: iScience. 2023 Nov 23;26(12):108546. doi: 10.1016/j.isci.2023.108546 (PMC10711493; doi:10.1016/j.isci.2023.108546)
Supplement: Document S1. Figures S1 and S2 [file mmc1.pdf]

iScience, Volume *26*

Supplemental information

Group size influences maternal provisioning

and compensatory larval growth in honeybees

Bin Han, Esmaeil Amiri, Qiaohong Wei, David R. Tarpy, Micheline K. Strand, Shufa Xu, and Olav Rueppell

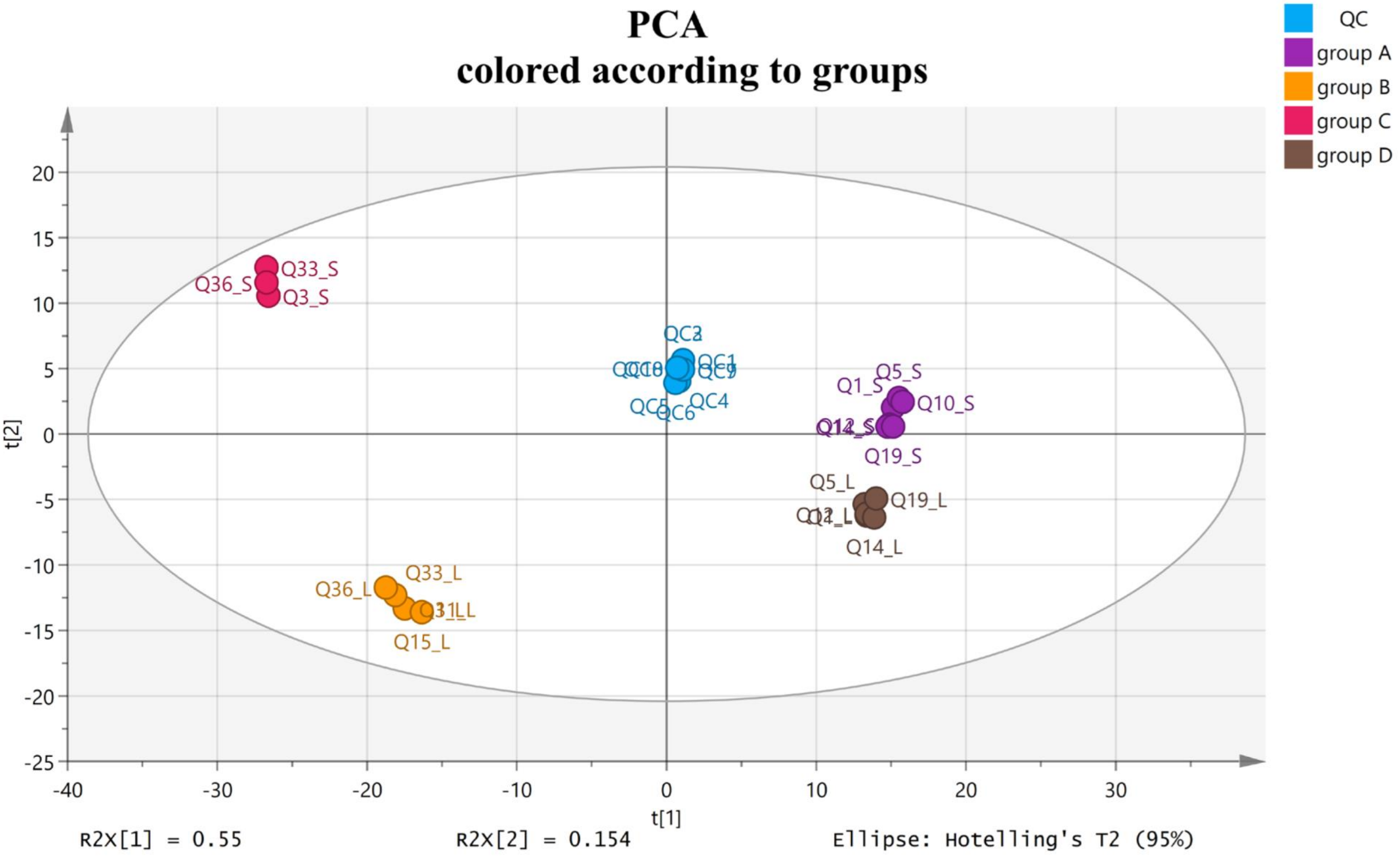

Supplemental Figure S1: Principal Component Analysis separates egg metabolomes according to egg size and sampling date, related to Figure 2. Group A = SJ (small colonies in July), Group B = LJ (large colonies in July), Group C = SA (small colonies in August), and Group D = LA (large colonies in August). Related to Figure 2.

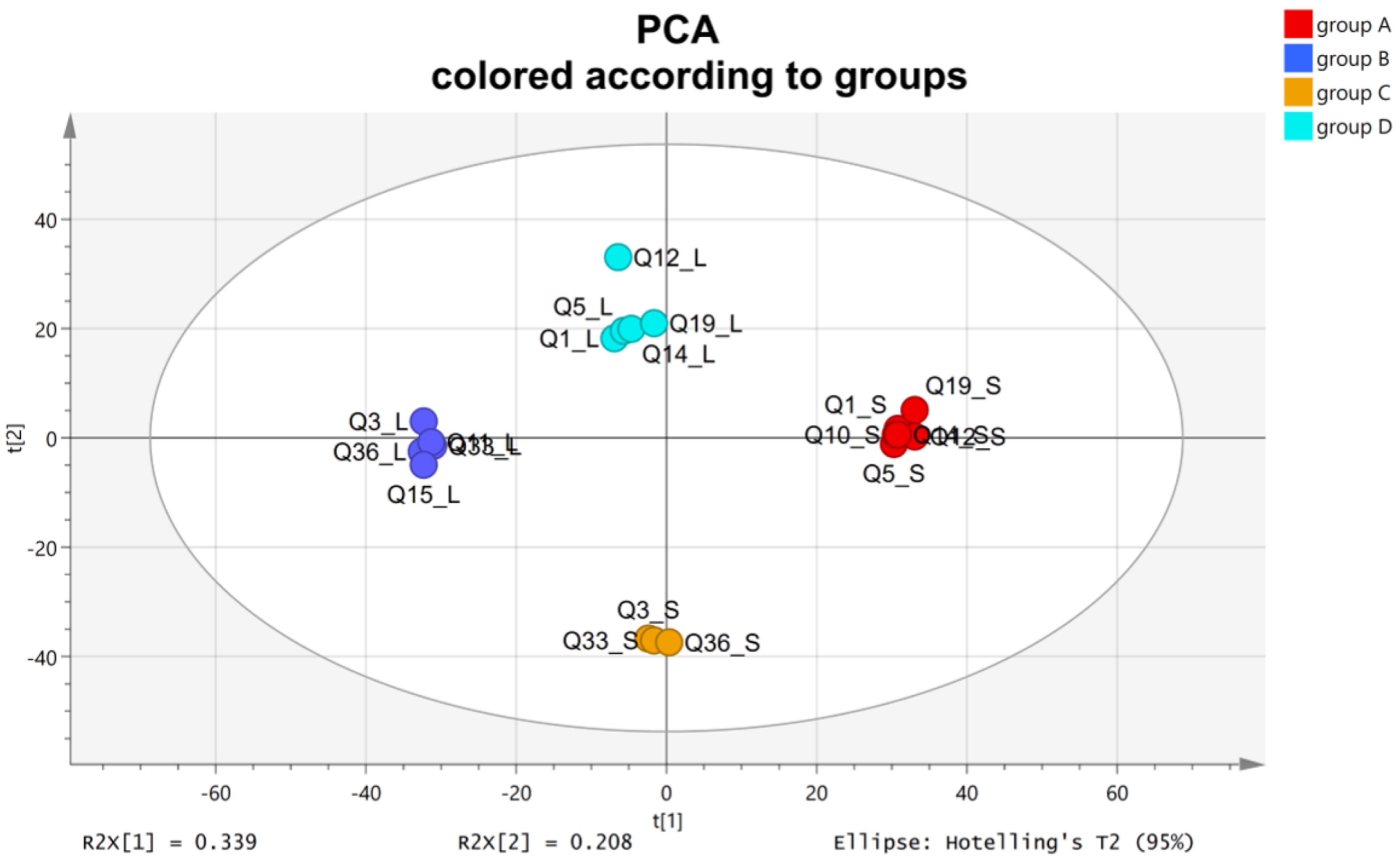

Supplemental Figure S2: Principal Component Analysis separates egg proteomes according to egg size and sampling date, related to Figure 3. QC = quality control, Group A = SJ (small colonies in July), Group B = LJ (large colonies in July), Group C = SA (small colonies in August), and Group D = LA (large colonies in August). Related to Figure 3.
